# Current Challenges and Advances in Preterm Birth

**DOI:** 10.3390/jcm10225257

**Published:** 2021-11-12

**Authors:** Alex Farr

**Affiliations:** Department of Obstetrics and Gynecology, Division of Obstetrics and Feto-Maternal Medicine, Medical University of Vienna, A-1090 Vienna, Austria; alex.farr@meduniwien.ac.at; Tel.: +43-1-40400-28210; Fax: +43-1-40400-28620

As the Guest Editor of this Special Issue, I would like to express my gratitude to all the authors who have contributed. I am thankful that we were able to accept a number of interesting articles of high quality among those received for publication.

An estimated 15 million infants worldwide are born before completing 37 gestational weeks, with resulting complications. Despite all efforts, preterm birth is still the predominant reason for an estimated one million neonatal deaths annually and a significant contributor to childhood morbidities. Modern perinatal and neonatal care has led to a substantial reduction in infant deaths and disability rates, yet preterm birth remains an ongoing challenge. It is a multifactorial event with a broad variety of causal factors, potentially involving multiple disciplines during antenatal and postnatal care.

Vaginal dysbiosis and infection are among the most frequent causes of preterm births. Early diagnosis is essential as it is well known that delayed treatment initiation increases the risk of preterm birth. In clinical practice, conventional diagnostic procedures are time consuming and require medical staff and equipment. As part of this Special Issue, Foessleitner et al. [1] reported high sensitivity and specificity of a point-of-care test to detect bacterial vaginosis during pregnancy, which could prevent preterm birth. In light of the high number of women who experience infection-related preterm birth, this public health intervention could be potentially groundbreaking.

Moreover, the importance of thorough antenatal care was confirmed in the article by Ferrillo et al. [2], who reported a high prevalence of preterm birth among women with poor oral health and vitamin D deficiency. Their results underline the need to constantly expand the knowledge on various causal factors.

In cases where infection has finally resulted in preterm rupture of membranes and subsequent preterm birth, culture-proven sepsis is currently considered the gold standard for the diagnosis of early-onset neonatal sepsis. As part of this Special Issue, Grill et al. [3] have evaluated their data by comparing culture-proven sepsis, clinical sepsis, and positive laboratory biomarkers as predictors of neonatal mortality. They found that clinical sepsis was almost four times more common than culture-proven sepsis and exhibited similar prediction ability for neonatal mortality and severe morbidity as culture-proven sepsis.

The cited articles confirm the multidisciplinary approach that is needed to adequately address preterm birth, offering healthcare professionals an insight into its complexity, thereby providing an understanding and promotion of various strategies. This approach is pursued in this Special Issue’s excellent review by Schachinger et al. [4], who summarized the current knowledge on musculoskeletal health-related disorders in preterm infants.

Finally, in our issue, Gorczyca et al. [5] have addressed the emerging problem of abnormally invasive placenta (AIP), as not only a result of the increasing rate of cesarean section throughout the last few decades, but also commonly resulting in iatrogenic preterm birth. The authors evaluated the extent of placental invasion and the presence of sonographic findings suggestive of AIP, reporting no detectable increase in invasiveness over the course of the second and third trimester of pregnancy. This is important for the surveillance of patients with AIP during pregnancy.

Taken together, the articles in this Special Issue have added to the increasing knowledge of preterm birth and its pathogenic role, and have provided a better understanding of established or newly developed approaches for this multifactorial event. I would, herein, like to thank all the authors and reviewers for their significant and highly appreciated contribution to our Special Issue, and for making it a valuable collection of studies that are very interesting to read and learn from.

Contributing to the understanding of preterm birth, which compromises the health of our future generations, is without a doubt a challenge worth investing.

## Data Availability

Not applicable.
